# The Genus *Conradina* (Lamiaceae): A Review

**DOI:** 10.3390/plants7010019

**Published:** 2018-03-11

**Authors:** Noura S. Dosoky, William N. Setzer

**Affiliations:** 1 Aromatic Plant Research Center, 615 St. George Square Court, Suite 300, Winston-Salem, NC 27103, USA; ndosoky@aromaticplant.org; 2 Department of Chemistry, University of Alabama in Huntsville, Huntsville, AL 35899, USA

**Keywords:** *Conradina*, essential oil, ursolic acid, cytotoxicity, antimicrobial, antileishmanial, phylogenetic analysis

## Abstract

*Conradina* (Lamiaceae) is a small genus of native United States (US) species limited to Florida, Alabama, Mississippi, Tennessee and Kentucky. Three species of *Conradina* are federally listed as endangered and one is threatened while two are candidates for listing as endangered. The purpose of the present review is to highlight the recent advances in current knowledge on *Conradina* species and to compile reports of chemical constituents that characterize and differentiate between *Conradina* species.

## 1. Introduction

*Conradina* A. Gray (Lamiaceae) is a small genus of morphologically distinctive, narrow-leaved, minty-aromatic shrubs endemic to the southeastern United States of America (USA) [1]. It consists of six to nine US native species. According to the Plant List database [2] and Integrated Taxonomic Information Species (ITIS), the acceptable number of species for the genus is only six (*Conradina canescens* A. Gray, *C. cygniflora* C.E. Edwards, Judd, Ionta & Herring, *C. etonia* Kral & McCartney, *C. glabra* Shinners, *C. grandiflora* Small and *C. verticillata* Jennison) while *C. brevifolia* Shinners, *C. montana* Small and *C. puberula* Small are considered synonyms of other species. The Missouri Botanical Garden, however, lists nine distinct species [3]. *Conradina* species are characterized by very dense hairs on their lower leaf surfaces and by a sharply bent corolla tube in the flowers [1,4]. Asa Gray established the genus *Conradina* in 1870, named for the American botanist Solomon White Conrad [5]. *Conradina* species grow well in xeric habitats with well-drained sandy soils. It is thought that *Conradina* may be a pioneer species in disturbed areas since it has the ability to colonize xeric disturbed habitats [1,5].

**Figure 1:**
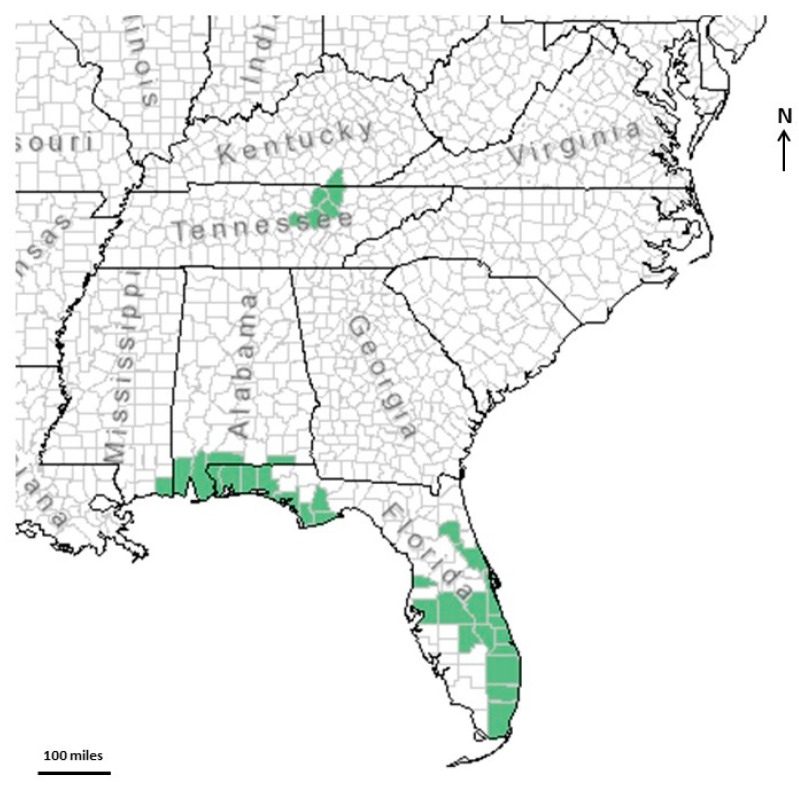
Locations of known populations of *Conradina* species.

Each species of *Conradina* occupies a separate geographical region [1,6]. Five of the species are endemic to Florida, one is native to west Florida, south Alabama, and south Mississippi, and one is endemic to north-central Tennessee and Kentucky (Figure 1). Due to habitat destruction from residential, commercial, and agricultural land conversions and their very restricted distribution, three of these species are on the United States Fish and Wildlife Service (USFWS) federal list as endangered (*Conradina brevifolia*, *C. glabra*, and *C. etonia*) [5], one as threatened (*C. verticillata*) [6] and two species are considered candidates for listing as endangered (*C. grandiflora* and *C. cygniflora*).

The aim of the present review is to summarize the literature in order to document secondary metabolites that characterize and differentiate between *Conradina* species. We shall also assess the reported biological activities of *Conradina* species, with particular focus on their phylogenetic affinities and anti-microbial, anti-leishmanial and cytotoxic properties.

## 2. Description

*Conradina* represents a group of evergreen, compact, perennial shrubs with virgate branches. Leaves are aromatic and needle-like with narrow, entire, revolute leaf-blades. Flowers are solitary or in a group of few in axillary cymes. The calyx is two-lipped; the lower lip has two long, narrow lobes while the upper lip has three short broad lobes. Corolla is white to purple in color, two-lipped; the lower lip has three lobes and usually dotted while the upper lip is erect and slightly concave.

*Conradina canescens* A. Gray (false rosemary) is the only species of this genus that is relatively common in its range and the most morphologically variable (Table 1)*.* It is native to a small area of west Florida and south Alabama and southern parts of Mississippi [7]. It occurs on sunny, dry sand soils on coastal dunes, in sand scrubs and in dry longleaf pineland ecosystems. It shows tolerance for the heat and humidity of the Southeast and is considered a drought-tolerant landscape plant that grows best in lean, sandy soils [8]. *C. canescens* is one of the indicator species for wooded dunes [9] and is an excellent candidate for restoration of coastal areas. It can be used for beach projects that require planting on the back side of primary dunes, or any side of secondary dunes. It has also been used in gardens as a groundcover as the branches can reach up to 1 m in spread. False rosemary was easily propagated in the University of Florida Institute of Food and Agricultural Sciences Extension using softwood stem cuttings from terminal shoots taken during the growing season.

Morphologically, *C. brevifolia* Shinners (short-leaved rosemary) is very similar to *C. canescens*, differing by its shorter leaves and greater number of flowers per axil, and it is also similar to the endangered *C. glabra* [1,5]. *C. brevifolia* was described as a new species by Shinners [4]. Many taxonomic reviews of *Conradina* have upheld *C. brevifolia* as a distinct species yet it is taxonomically questionable [1,6,10,11]. Some taxonomists are still uncertain that *C. brevifolia* is a true different species and therefore they treat it as a synonym to *C. canescens. C. brevifolia* has a very restricted range in the middle of the Lake Wales Ridge in Central Florida [12,13,14] and has been listed as a federally endangered species in 1993 [5]. Habitat destruction for agricultural and residential purposes is the main threat to this species.

*C. etonia* Kral & McCartney (Etonia rosemary) was discovered in the Etonia Scrub in Putnam County, Florida in September 1990 and described as a new species in 1991 [6]. *C. etonia* is similar to *C. grandiflora* in general appearance and the flowers are large and quite similar but the leaves of *C. etonia* are broader than *C. grandiflora* and have clearly visible lateral veins on the lower surface [1,15]. *C. etonia* is found in very limited areas of deep white sand scrub dominated by sand pines (*Pinus clausa*) and scrubby oaks (*Quercus* spp.) on dry soils. Its entire known range is within a subdivision of Putnam County containing streets and a few residences, which make it the most narrowly distributed species of *Conradina* [6]. *C. etonia* was listed as a federally endangered species in July 1993 [5]. The latest survey of Etonia rosemary counted about eight subpopulations on Etonia Creek State Forest containing fewer than 1000 total individuals [16]. The major threats facing *C. etonia* include habitat loss due to development projects for residential housing, horticultural collection, hurricane damage and invasive species or lack of natural processes, such as fire [17,18]. Because *C. etonia* requires light but can tolerate the light shade of openings the in scrub, fire suppression is one of the major limiting factors as it results in over-shading by overstory scrub vegetation rendering the habitat less favorable for *C. etonia* [18].

*C. grandiflora* Small (large-flowered rosemary) is endemic to the scrub habitat of Florida’s east coast between Daytona Beach and Miami as well as near Orlando and Okeechobee County [5]. A regime of frequent fires benefits *C. grandiflora* because it cannot tolerate shade and requires an open sunny habitat. *C. grandiflora* is a candidate for listing as an endangered species but of a lower priority than other *Conradina* species [5]. Loss of the Florida scrub habitat is the major threat to this species.

*C. glabra* Shinners (Apalachicola rosemary) was described in a sand hill community east of the Apalachicola River in Liberty County, Florida in 1962 [1,4]. This species is very narrowly distributed and restricted to Liberty County, Florida. Out of its seven known locations, six *C. glabra* subpopulations (artificially divided by the Florida gas transmission pipeline) are remaining, all of which are located on private silvicultural lands and subject to much disturbance. Its other historical locations were converted to pine plantations, and can no longer support this species. Because of homozygosity and inbreeding, many of flowers of *C. glabra* are male sterile, and the stamens are very malformed [1]. It was added to the federal list of endangered species in 1993 [5,19,20], primarily due to habitat loss and modification of forestry practices. There is no record of how abundant *C. glabra* was before its habitat was altered because the silviculture operations began before its discovery.

*C. verticillata* Jennison (Cumberland rosemary) is the only species of this genus that is not found in Florida. The species was established in 1933 [21]. It is a rare species, existing only as three small subpopulations in Tennessee and one subpopulation in Kentucky [22]. *C. verticillata* is found only in boulder bars, cobble bars, sand bars and gravel bars in close proximity to rivers on the Cumberland Plateau of north-central Tennessee and southeastern Kentucky [22]. The preferred habitat conditions include open to slightly-shaded area, moderately deep well-drained sandy soil, periodic flooding, and topographic features like narrow channels or depressions on gravel bars. *C. verticillata* is the only species that is triploid (*n* = 3) and accordingly has a lower ability to reproduce and disperse sexually and greatly reduced seed germination rates. The fresh seeds of *C. verticillata* are physiologically dormant and require cold stratification to germinate [23]. It has been on the federal list of threatened species since 1991 [24]. The major threats facing *C. verticillata* are habitat destruction, general deterioration of water quality, competition and shading by woody plants.

In 2009, analysis of patterns of genetic structure based on microsatellites in *Conradina* led to the identification of *C. cygniflora* Edwards, Judd, Ionta & Herring [25]. *C. cygniflora* can only be found in Dunns Creek state park in south-central Putnam County, Florida. It occupies nine tightly-clustered sites that probably form around two to four self-sustaining subpopulations. *C. cygniflora* carries several unique morphological characters that distinguish this species from all other described *Conradina* species including thin-walled unicellular hairs, epidermis features, and larger calyces. Due to its exceptionally limited geographic distribution and low number of individuals, *C. cygniflora* is considered a candidate for listing as federally endangered [25].

There is very little information in the literature about *C. montana* and *C. puberula*. *C. montana* Small was reported in sandy woods and ravines, Appalachian plateau near Rugby, Tennessee while *C. puberula* Small was observed in pine-lands, northern Gulf coast region, Florida [26,27]. Some taxonomic discrepancies surround *C. montana* and *C. puberula* since some experts treat both species as synonyms of *C. verticillata* and *C. canescens*, respectively.

## 3. Phylogenetic Studies

*Conradina* is a member of Lamiaceae and belongs to a clade of New World Mentheae. The closely related genera that share similar morphology and habitat preference with *Conradina* include *Dicerandra* spp., *Piloblephis* spp., *Stachydeoma* spp., and the woody, southeastern U.S. species of *Clinopodium* such as *C. ashei, C. georgianum, C. coccineum*, and *C. dentatum* [28]. Despite the fact that *Conradina* species are morphologically distinguishable, there are two problems about species status based on morphology. First, *C. brevifolia* (endangered species) shares a lot of morphological characteristics with *C. canescens* (relatively widespread in its range). Second, populations of *Conradina* in Santa Rosa County, Florida, have morphological characteristics in common with both *C. glabra* (endangered species) and *C. canescens.* Due to the endangered status of four *Conradina* species, it is important to resolve the species relationships, define species limits and elucidate the evolutionary processes in order to help with conservation plans. *Conradina* was subjected to several phylogenetic analyses using four data partitions: plastid DNA, internal transcribed spacer (ITS), and two members of the GapC gene family [29,30,31]. These analyses did not resolve species relationships in *Conradina*. In agreement morphological evidence, ITS results supported a monophyletic *Conradina* while plastid DNA results did not. Some *Conradina* populations showed moderate levels of inbreeding, but inbreeding does not seem as a major factor. Because previous analyses failed to resolve species relationships separately, combined analyses were carried out but the results still did not resolve the relationships within *Conradina*. Studies using genotype data from quickly evolving microsatellite loci suggested that both ancient interspecific hybridization and incomplete lineage sorting of ancestral polymorphism have likely occurred in *Conradina* and that based on the patterns of genetic structure (which corresponds to species bounderies), *Conradina* species have diverged genetically from one another (nonmonophyly) despite having unique distribution patterns and distinguishing morphological characteristics [31].

## 4. Essential Oils and Non-Volatile Components

### 4.1. Essential Oils

**Figure 2:**
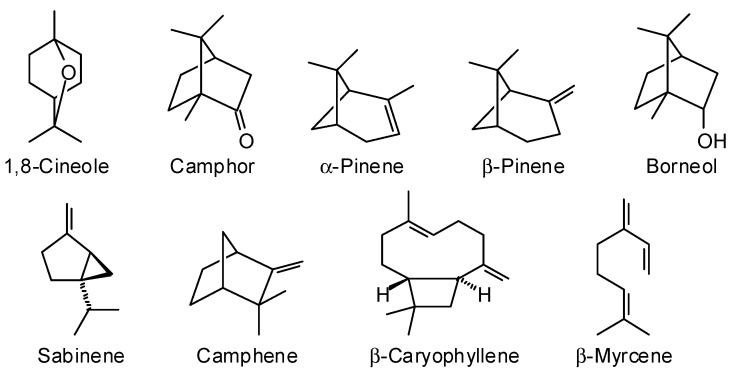
Chemical structures of common components found in six species of *Conradina*.

The essential oils obtained from the aerial parts of *Conradina* spp. are rich in terpenes, terpenic aldehydes and ketones, and terpenic alcohols [32,33]. Terpenes released from *Conradina* are allelopathic, and are believed to help prevent wildfires [34,35] and to protect against insects [32]. The main components quantified in the essential oils of *Conradina* species with the respective percentages (%) are summarized in Table 2. The essential oil of *C. cygniflora* has not been studied yet. 1,8-Cineole and camphor are the major components of *C. canescens*, *C. brevifolia, C. glabra and C. verticillata* [7]. The common components found in all *Conradina* species (excluding *C. cygniflora*) share a terpenoid skeleton but vary in cyclization and oxygenation (Figure 2). These common components include 1,8-cineole (bicyclic monoterpene ether), camphor (bicyclic monoterpene ketone), α-pinene (bicyclic monoterpene), β-pinene (bicyclic monoterpene), β-myrcene (acyclic monoterpene), borneol (bicyclic monoterpene alcohol), sabinene (bicyclic monoterpene), camphene (bicyclic monoterpene), and β-caryophyllene (bicyclic sesquiterpene).

### 4.2. Nonvolatile Components

**Figure 3:**
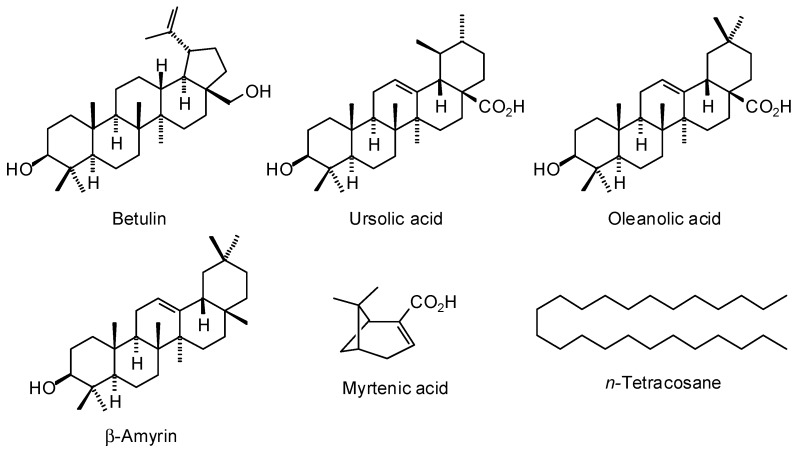
Chemical structures of nonvolatile components isolated from *Conradina canescens*.

*Conradina* is a reservoir of several important bioactive molecules. However, the chemical studies on *Conradina* are very limited in number. Six compounds were separated from the chloroform extract of *C. canescens* leaves (Figure 3): ursolic acid, betulin, β-amyrin, myrtenic acid, *n*-tetracosane, and oleanolic acid [36]. *C. canescens* leaves are considered a natural source for ursolic acid [37].

## 5. Biological Activities

### 5.1. Allelopathic Activity

The term allelopathy describes the chemical interactions between plants where one plant interferes with the germination and growth of another plant. Allelopathic compounds are thought to enter the surrounding environment via volatilization, leaching with rain, and decomposition of plant litter, thereby inhibiting the growth of competitors or species that may threaten the plant’s survival. In this manner, the plant may have an ecological role in its ecosystem by affecting plant spacing, succession, and community composition [35]. Terpenoids, especially monoterpenoids, are considered one of the 14 allelochemical classes [38,39] that play an important role in the interactions between plants [40,41]. *C. canescens* is thought to play a significant ecological role in maintaining a healthy scrub ecosystem by inhibiting the germination of native grasses [42]. It was suggested that by inhibiting germination of the grasses, the development of the more fire-prone sand hill ecosystem is prevented, and the scrub ecosystem is maintained [35]. Because detailed knowledge of allelopathic actions in natural plant communities can provide excellent models for new strategies in developing highly selective herbicides, the allelopathic effect of *C. canescens* has been a subject of several studies [34,35,43]. Water washes of fresh *C. canescens* leaves were reported to have strong inhibitory effects on germination and growth of *Schizachyrium scoparium* [34,43]. The essential oil of *C. canescens* and purified ursolic acid remarkably inhibited the germination of *Lactuca sativa* and *Lolium perenne* [37]. The phytotoxic activity of *C. canescens* oil can be attributed to the high concentration of 1,8-cineole which can inhibit the seed germination and seedling growth of lettuce in a dose-dependent manner [44,45,46,47,48] by strongly inhibiting mitochondrial respiration and all stages of mitosis [49]. The presence of significant quantities of ursolic acid suggests that this compound might contribute to the strong allelopathic effect of *C. canescens* [50,51]. Ursolic acid is thought to act as a natural detergent by leading water-insoluble monoterpenes to form micelles, rendering them water-soluble, thereby enhancing their ability to leach into rainwater for delivery into the soil [42,50]. Ursolic acid helps to co-solubilize the allelopathic monoterpenes in water to be more effective [52].

### 5.2. Antibacterial and Antifungal Activity

The essential oil of *C. canescens* has no antibacterial activity but has a slight antifungal activity against *Botrytis cinerea* [33] while the crude extracts showed good antifungal activity against *Botrytis cinerea* [36]. Ursolic acid, the major component in *C. canescens* leaves was reported to have selective antibacterial activity against *S. aureus* [36,53] while its isomer, oleanolic acid, was active against *S. aureus* and *Pseudomonas aeruginosa*. Interestingly, isolated *n*-tetracosane showed antibacterial activity against *Staphylococcus aureus* and *S. epidermidis* and antifungal activity against *Aspergillus niger* and *B. cinerea*. Isolated myrtenic acid was active against *S. aureus*, *A. niger*, *B. cinerea* and *Candida albicans* [36].

### 5.3. Cytotoxic Activity

The essential oil of *C. canescens* has no significant in vitro cytotoxic activity against the human breast tumor cell lines MCF-7 and MDA-MB-231 [33]. The crude chloroform extract of *C*. *canescens* showed a significant cytotoxic activity against MCF-7 (human breast tumor), MDA-MB-231 (human breast tumor) and 5637 (human bladder tumor) cell lines which is attributed to the presence of betulin and ursolic acid, which when tested individually showed strong effects against all tested cell lines [36].

### 5.4. Antileishmanial Activity

The crude extracts have promising antileishmanial activity against promastigotes and intracellular amastigotes of *Leishmania amazonensis*. However, the dichloromethane extract had some cytotoxicity on the host cells. The antileishmanial activity of the nonpolar extract was attributed to the presence of ursolic acid and betulin being the major constituents [36].

## 6. Conclusions

This review summarizes the current status, distribution, chemical value and biological studies on genus *Conradina*. There is still some debate about the exact number of *Conradina* species as well as the classification/taxonomy of this genus and more studies, especially genetic studies, are still needed. The available information increases the importance of protecting *Conradina* from extinction. Future studies on this genus may provide valuable information on the mechanism of evolution.

## Figures and Tables

**Table 1:** Morphological characteristics of *Conradina* species.

Species	Morphological Characteristics	References
*C. canescens*	Small shrub, up to 1 m high. Leaves are 7 to 20 mm long, mostly longer than the internodes. Leaf blades are pubescent on both sides. One to three flowers per axil, lower corolla-lip 8–10-mm long; lateral lobes longer than wide. Calyx-tube hirsute or villiou-hirsute.	[5]
*C. brevifolia ^a^*	Small shrub, up to 1 m high. Leaves are short fleshy 6.0 to 8.2 mm long, mostly shorter than the internodes, covered with short downy hairs and many tiny glands on the upper side. One to six lavender flowers per axil.	[5,11]
*C. etonia*	Straight slender shrub, about 1.5 m high. Leaves have hairy, veiny, glandular blades 1.5–3 cm long and 3–9 mm wide with tightly rolled edges. Three to seven flowers per axil. Pink to lavender in color with darker dotted lower petal.	[5,15,18]
*C. glabra*	Small shrub, about 80 cm high but some individuals reach up to 2 m. Leaves are opposite, up to 1.5 cm long, hairless on the upper surface. Two to three flowers per axil. Corolla is 1.5–2 cm long, white to pale lavender in color with a band of purple dots on the lower lip.	[5,19,20]
*C. grandiflora*	Erect shrub, 1.5–2.0 m high, with hairy branches and twigs. Leaves are hairy, glandular, up to 1.5 cm long. Year-round hairy lavender flowers with darker lavender spots, lower lip is 12–15 mm long with lateral lobes longer than wide. This species has the largest flowers of genus *Conradina*.	[5]
*C. verticillata*	Erect shrub, 0.5 m high with reclining branches. Leaves are about 2.5 cm long, very narrow, and arranged in tight bunches that appear as whorls around the stems. Flowers are 2.5 cm long, purple to white and borne in leaf-like clusters of bracts at the ends of the stems.	[24]
*C. montana ^b^*	Short shrub less than 0.5 m high with diffuse branches. Leaves are narrowly linear, 5–16 mm long. Leaf blades are glabrous on the upper surface. Minute flowers with corolla 3.5–4 mm long. Calyx-tube hirsutulous.	[23]
*C. puberula ^a^*	Short shrub of about 0.5 m high with numerous slender branches. Leaves are narrowly linear but strongly revolute and clavate, 12–20 mm long. Calyx-tube minutely canescent, 5–7 mm long. Flowers appear in racemes of 2–6 per axil, with corolla 4–5 mm long.	[26,27]
*C. cygniflora*	Virgate shrub up to 1 m high, branches are erect to spreading, internodes 5–43 mm long. Leaves persistent, appearing fascicled- verticillate; narrowly obovate, 9–33 mm long. The abaxial leaf surface is densely-covered by simple unicellular hairs. Cymes carry 1–5 subsessile flowers, densely pubescent, 1.3–12.5 mm long. Large calyx of 8.5–11 mm long; densely covered with simple hairs, upper lip upcurved, 3.6–4.4 mm long, lower lip 4.3–5.5 mm long. Corolla strongly bilabiate, 20–29 mm long, lavender, shading to white in throat, with purple spots; abaxial surface of upper lip darker lavender.	[25]

^a^ *C. brevifolia and C. puberula* are sometimes treated as synonyms to *C. canescens*, ^b^ *C. montana* is sometimes thought as a synonym to *C. verticillata.*

**Table 2:** Chemical composition of essential oils of *Conradina* species.

Species	Major Oil Components (%)	Unique Component(s)	Reference
*C. brevifolia ^a^*	Camphor (9.7–17.54%) and 1,8-cineole (1.97–4.86%)	α- and β-farnesene	[7]
*C. canescens ^a^*	Camphor (0.27–23.64%), 1,8-cineole (0.17–3.34%), cis-pinocamphone (0–8.74%)	none	[7]
*C. canescens ^b^*	1,8-cineole (5.2–25.2%), camphor (5.7–8.0%), α-pinene (3.2–5.6%), *p*-cymene (3.3–5.9%), *cis*-pinocamphone (1.3–5.5%), myrtenal (5.2–8.1%), myrtenol (3.4–9.2%), verbenone (4–4.5%), and myrtenyl acetate (5.0–5.4%)	-	[33]
*C. etonia ^a^*	Camphor (30.55–35.65%), limonene (3.77–6.33%), camphene (2.92–3.75%), and β-caryophyllene (2.95–6.54%)	β-elemene, 4-carene and α-terpineol	[32]
*C. glabra ^a^*	1,8-cineole (2.38–7.34%) and camphor (11.78–15.88%)	Dolcymene and bornyl acetate	[7]
*C. grandiflora ^a^*	β-pinene (4.38–5.81%) and β-cubebene (1.95–6.56%)	Calarene and β-pinone	[7]
*C. verticillata ^a^*	1,8-cineole (3.15–3.78%) and camphor (5.81–8.35%)	Germacrene B and 2,5,6-trimethyl-1,3,6-heptatriene	[7]
*C. cygniflora*	Data not available	N/A	N/A

^a^ Essential oil obtained by solvent extraction, ^b^ essential oil obtained by hydrodistillation.
